# Associations between the Self-Reported Likelihood of Receiving the COVID-19 Vaccine, Likelihood of Contracting COVID-19, Discrimination, and Anxiety/Depression by Sexual Orientation

**DOI:** 10.3390/vaccines11030582

**Published:** 2023-03-02

**Authors:** David Adzrago, Cameron K. Ormiston, Saanie Sulley, Faustine Williams

**Affiliations:** 1 Division of Intramural Research, National Institute on Minority Health and Health Disparities, Two White Flint North, Rockville, MD 20852, USA; 2 National Healthy Start Association, 1325 G Street, Washington, WA 20005, USA

**Keywords:** vaccine, COVID-19 pandemic, sexual minority, discrimination, mental health

## Abstract

There is limited evolving literature on COVID-19 vaccine uptake and its barriers among sexual minority populations (lesbian, gay, bisexual, transgender, and queer [LGBTQ]), despite their increased COVID-19 risk factors. We assessed the differences in intention to receive the COVID-19 vaccine by self-reported likelihood of contracting COVID-19, anxiety/depression, discrimination frequency, social distancing stress, and sociodemographic factors across sexual orientation. An online national cross-sectional survey was conducted in the United States between 13 May 2021, and 9 January 2022, among adults aged ≥18 (n = 5404). Sexual minority individuals had a lower intention of receiving the COVID-19 vaccine (65.62%) than heterosexual individuals (67.56%). Disaggregation by sexual orientation, however, showed that gay participants had a higher intention of COVID-19 vaccination (80.41%) and lesbian (62.63%), bisexual (64.08%), and non-heterosexual, non-LGB sexual minority (56.34%) respondents had lower intentions of receiving the COVID-19 vaccine than heterosexual respondents. Sexual orientation significantly moderated the association between the perceived likelihood of receiving the COVID-19 vaccine and the self-reported likelihood of contracting COVID-19, anxiety/depression symptoms, and discrimination. Our findings further underline the importance of improving vaccination efforts and access among sexual minority individuals and other vulnerable groups.

## 1. Introduction

Since the beginning of the COVID-19 pandemic, marginalized communities, communities of color, and other minoritized groups in the United States (US) have faced a disproportionate burden of COVID-19 risk, morbidity, and mortality [1,2,3]. Among these disproportionately at-risk groups are lesbian, gay, bisexual, transgender, and queer (LGBTQ) communities [4,5,6,7]. LGBTQ persons have a greater incidence asthma, cancer, diabetes, heart disease, substance use, and mental health symptoms—all of which are associated with minority stressors (e.g., discrimination, stigma) and predispose them to COVID-19 morbidity and mortality [4,8,9,10,11]. Furthermore, it has been reported in studies conducted in the US that LGBTQ adults were more likely to experience worsening physical and mental health compared to non-LGBTQ adults during the pandemic [6,8,12]. For instance, a study utilizing a national sample of US adults found loneliness, stress, psychological distress, and quality of life were worse among sexual minority individuals compared with non-sexual minority persons [8].

The increased rate of COVID-19 risk factors among LGBTQ communities is further compounded by already-existing and sustained financial, social, and structural barriers and inequities that act against sexual and gender minority individuals. Prior to the pandemic, LGBTQ communities reported poorer healthcare access and quality (e.g., lack of insurance, financial constraints, medical mistrust due to discrimination), greater discrimination and stigma, and higher rates of poverty, unemployment, incarceration, and housing instability compared to non-LGBTQ people [9,13,14]. During the pandemic, LGBTQ adults have experienced greater job loss, financial instability, lack of basic needs, social isolation, and barriers to healthcare access [6,12,15]. LGBTQ individuals with intersectional identities, such as those who are also part of a racial/ethnic minority group, are at even greater risk [4,15,16].

Despite these alarming trends, LGBTQ communities have been given little attention in terms of pandemic surveillance and research, and up until October 2021—more than a year after the first COVID-19 case was reported—no public reporting at the local, state, and federal level had been done on the impact of COVID-19 on LGBTQ communities [17,18]. At the time of writing this paper (October 2022), there are still no data on COVID-19 morbidity and mortality among LGBTQ communities. Evidently, the public health response against COVID-19 in LGBTQ communities has been inadequate, highlighting the cisgenderism and structural homophobia, transphobia, and biphobia still present in our system, leaving many LGBTQ communities feeling forgotten and neglected and drawing comparisons to the 1980s’ HIV/AIDS epidemic response [18,19]. Ultimately, the constellation of COVID-19 risk factors, systemic and individual barriers to well-being, and socioeconomic inequities indicate an increasingly salient need for more research and action. This is especially the case when considering that these factors and barriers are known to adversely affect health behaviors and may influence vaccine coverage among LGBTQ communities [9,13,20,21].

Despite COVID-19 vaccine interest and confidence being higher among LGBTQ individuals, disparities have been noted in the uptake of and access to COVID-19 vaccination [9,22]. Reported barriers include the absence of LGBTQ representation in vaccine education, the medical field, and vaccination efforts, a lack of LGBTQ-affirming clinical spaces, historical and current medical trauma (e.g., misgendering, dead-naming, discrimination, stigma, neglect of LGBTQ communities during the HIV epidemic), medical mistrust, and fear of abuse, violence, and mistreatment [20]. For instance, medical mistrust and stigma have been linked to lower levels of COVID-19 vaccine acceptance among LGBTQ men and transgender women [21]. Additionally, vaccine coverage may be further at-risk among LGBTQ communities of color, given these communities face both anti-LGBTQ and racial/ethnic discrimination, stigma, and medical abuse.

Presently, knowledge on COVID-19 vaccination uptake and attitudes among sexual minority populations is still developing. According to the Theory of Planned Behavior (TPB), an individual’s vaccine intention is likely to be influenced by their attitudes, subjective norms and beliefs, or perceived behavioral control over COVID-19 vaccination [23]. Furthermore, vaccination intentions can be modified by sociodemographic and mental health factors [23]. Likewise, McNaghten et al. [13] reported higher levels of vaccine confidence and vaccination among LGB adults compared to non-LGB persons (as of 30 October 2021), and barriers to vaccine uptake unique to sexual minority communities continue to persist [20]. Ultimately, given the greater vulnerability to COVID-19, barriers to healthcare access among the LGBTQ community, and the relative dearth of data on LGBTQ populations during the COVID-19 pandemic, understanding COVID-19 preventive behaviors and experiences among sexual minority individuals are of paramount importance.

This study aims to provide a more nuanced understanding of COVID-19 vaccination intention patterns, examining between- and within-group differences by sexual orientation. Using a national survey of US adults, we aimed to (1) estimate the prevalence of intention to receive the COVID-19 vaccine by sexual orientation; (2) assess the differences in intention to receive COVID-19 vaccination by self-reported likelihood of contracting COVID-19, anxiety/depression symptoms, discrimination frequency, social distancing stress, and sociodemographic factors across sexual orientation; and (3) determine if sexual orientation moderates the association between intention to receive COVID-19 vaccine and perceived likelihood of contracting COVID-19, anxiety/depression, and discrimination frequency.

## 2. Methods

### 2.1. Study Design and Samples

This US national cross-sectional survey was conducted between 13 May 2021 and 9 January 2022 among adults aged 18 years or older. The National Institutes of Health conducted it to assess COVID-19 vaccination perceptions, perceived likelihood of contracting COVID-19, social distancing stress, mental health, discrimination frequency, and participants’ sociodemographic characteristics. A total of 5938 participants completed the survey that Qualtrics LLC distributed. After data cleaning, 5413 samples were achieved. Participants (n = 5404) from whom complete data was given on responses to a question about the perceived likelihood of receiving the COVID-19 vaccine were included in the analysis. Actual vaccination status was not assessed with the perceived likelihood of receiving the COVID-19 vaccine.

### 2.2. Measures

The perceived likelihood of receiving the COVID-19 vaccine was the dependent variable and was measured using the question, “Now that we have a Coronavirus/COVID-19 vaccine available, have you been/planning to be vaccinated?” (Not at all likely, slightly likely, moderately likely, very likely, and extremely likely). We recategorized the response options into two categories: not at all likely and likely (i.e., slightly likely, moderately likely, very likely, and extremely likely). The recategorization was based on the stratification of sexual orientation and small cell counts or samples within subgroups.

Self-reported sexual orientation, the likelihood of contracting COVID-19, anxiety/depression symptoms, discrimination frequency, or social distancing stress were the main independent variables. Sexual orientation is a spectrum and includes but is not limited to heterosexual, lesbian, gay, bisexual, queer, pansexual, and asexual [24]. However, due to sample size, we dichotomized sexual orientation into heterosexual and sexual minority (lesbian, gay, bisexual, or something else [write-in option]). Perceived likelihood of contracting COVID-19 was determined by asking the participants whether they feel they will contract COVID-19 based on their overall self-rated health (not at all likely or likely). Discrimination frequency was measured based on how often (never, about once a month, about once a week, 2 to 3 times a week, and daily or almost daily) the participants experienced any discrimination during the COVID-19 pandemic. To determine social distancing stress status, the participants were asked, “How stressful has socially distancing been for you?” Response options, which included very stressful, somewhat stressful, a little stressful, or not at all stressful, were recategorized into two groups: (1) stressful if the participants selected very stressful, somewhat stressful, or a little stressful; and (2) not at all stressful.

Four questions were used to derive anxiety/depression symptoms, which reflect the Patient Health Questionnaire-4 (PHQ-4) scale [25,26]. The four questions entail how often (not at all = 0, several days = 1, more than half the days = 2, or nearly every day = 3) over the last two weeks the participants have been bothered by (1) feeling nervous, anxious or on edge, (2) not being able to stop or control worrying, (3) feeling down, depressed or hopeless, and (4) little interest or pleasure in doing things [25,26]. The total score from the questions or for the PHQ-4 range from 0–12; minimal/negative (0–2), mild (3–5), moderate (6–8), and severe (9–12).

The sociodemographic factors that may be related to perceived likelihood of receiving the COVID-19 vaccine are age in years (18–25, 26–34, 35–49, 50 or more), gender identity (Man, Something else [write-in]/non-binary/transgender, Woman), race/ethnicity (Another race/ethnicity [i.e., American Indian, Alaskan Native, Middle Eastern, Pacific Islander], Asian, Black/African American, Hispanic/Latino, White), level of education completed, and annual household income. Thus, this study examined these variables as covariates of the perceived likelihood of receiving the COVID-19 vaccine.

### 2.3. Statistical Analyses

To analyze the data, we first estimated the prevalence of the perceived likelihood of receiving the COVID-19 vaccine based on sexual orientation (heterosexual, lesbian, gay, bisexual, and something else) using Chi-Squared tests. Second, we performed descriptive and bivariate statistics to describe the prevalence of and differences in the perceived likelihood of receiving the COVID-19 vaccine by sociodemographic characteristics, perceived likelihood of contracting COVID-19, anxiety/depression, discrimination frequency, and social distancing stress across sexual orientation (heterosexual vs. sexual minority). Chi-Squared tests were used to determine the differences. Furthermore, we conducted moderation analyses to determine whether sexual orientation moderates the association between the perceived likelihood of receiving the COVID-19 vaccine and anxiety/depression, discrimination frequency, perceived likelihood of contracting COVID-19, or social distancing stress, respectively.

Finally, to evaluate the association between the perceived likelihood of receiving the COVID-19 vaccine and anxiety/depression, discrimination frequency, perceived likelihood of contracting COVID-19, and social distancing stress, adjusting for the sociodemographic characteristics based on sexual orientation (heterosexual vs. sexual minority), multivariable logistic regression models were utilized. The models were conducted at an alpha level of 0.05, 95% confidence intervals (CIs), and adjusted odds ratios (AORs). We examined multicollinearity among the independent variables, but no significant multicollinearity issues were observed because the mean-variance inflation factor (VIF) of 1.15 was less than the 10 thresholds for serious multicollinearity [27]. The analyses were performed using STATA/SE version 16 [28]. We applied listwise deletion method to handle the missing values. This study received Institutional Review Board (IRB) approval on 23 December 2020 (IRB #000308) as an exempt protocol from the IRB—Human Research Protections Program—Office of Human Subjects Research Protections.

## 3. Results

**Figure 1:**
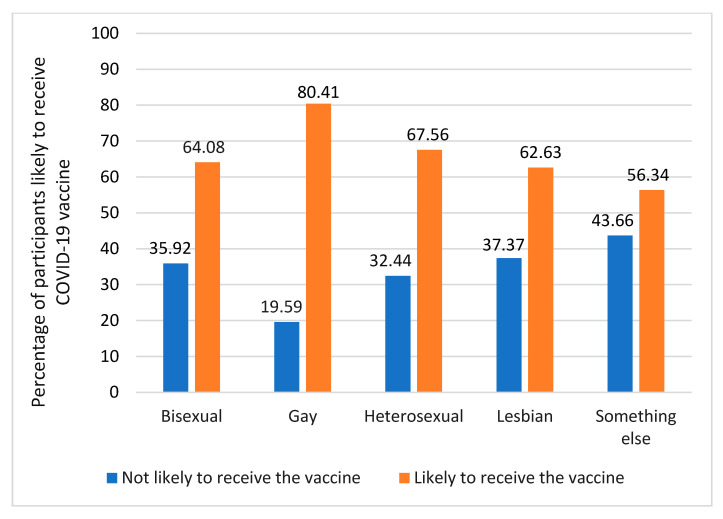
Prevalence of self-reported likelihood of receiving COVID-19 vaccine by sexual orientation.

Figure 1 shows the prevalence of the perceived likelihood of receiving the COVID-19 vaccine by sexual orientation. There was a statistically significant difference in the perceived likelihood of receiving the COVID-19 vaccine among the sexual orientation groups (chi2[4] = 14.05, *p* = 0.007). Gay individuals had a higher prevalence of the likelihood of receiving the vaccine, followed by those who identified as heterosexual, bisexual, lesbian, and something else.

Table 1 presents the descriptive and bivariate analysis results based on sexual orientation. The highest proportion of heterosexual participants were aged 35–49 years (23.06%), women (62.85%), White (43.26%), had college or higher education (39.89%), had an annual household income of $75,000 or more (27.40%), perceived likelihood of contracting COVID-19 as likely (68.55%), and indicated social distancing as stressful (68.16%). A proportion of the participants also experienced mild (21.86%), moderate (11.98%), and severe (10.75%) anxiety/depression symptoms. The participants experienced discrimination about once a month (15.52%), once a week a week (10.16%), 2–3 times a week (9.78%), and daily or almost daily (11.95%). About 67.56% of the heterosexual participants perceived receiving the COVID-19 vaccine. The perceived likelihood of receiving the COVID-19 vaccine varied among subgroups of heterosexual participants. The highest prevalence of the individuals that reported that they were likely to receive the COVID-19 vaccine was among those who were 50 years or older (87.68%), Asian (81.94%), had college or higher education (79.16%), had an annual household income of $75,000 or more (80.48%), or reported being likely to contract COVID-19 (69.75%).

Sexual minority participants were mostly 18–25 years (15.62%), women (59.72%), White (37.15%), had some college/vocational or technical school education (30.73%), annual household income of less than $25,000 (29.95%), a likely perceived likelihood of contracting COVID-19 (67.65%), and reported social distancing as stressful (74.13%). Approximately 25.04%, 24.16%, and 20.99% experienced mild, moderate, and severe anxiety/depression symptoms, respectively; 16.32%, 14.76%, and 18.92% experienced discrimination about once a month, once a week or 2–3 times a week, and daily or almost daily, respectively. The prevalence of participants being likely to receive the COVID-19 vaccine was 65.62% and was highest among those who were 35–49 years (91.78%), men (74.03%), Asian (84.00%), had college or higher education (84.88%), or had an annual household income of $75,000 or more (79.17%).

### 3.1. Moderation Analysis

There was a significant interaction between sexual orientation and social distancing stress (chi2[2] = 6.40, *p*= 0.041). However, the model was not significant (chi2[3] = 7.50, *p* = 0.058) after including the three terms (main effects and the interaction terms).

**Figure 2:**
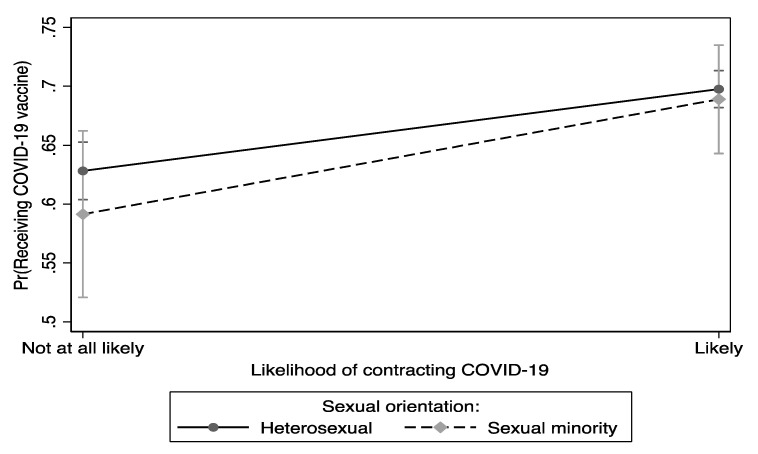
Differences in the perceived likelihood of receiving the COVID-19 vaccine between and within sexual orientation and self-reported likelihood of contracting COVID-19.

Figure 2 presents whether sexual orientation moderates the association between the perceived likelihood of receiving the COVID-19 vaccine and the likelihood of contracting COVID-19. There was a significant interaction between sexual orientation and the likelihood of contracting COVID-19 (chi2[2] = 28.33, *p* < 0.001). Heterosexual and sexual minority individuals who had a greater perceived likelihood of contracting COVID-19 had the highest probability of being likely to receive the COVID-19 vaccine compared to their counterparts who reported their likelihood of contracting COVID-19 as not likely.

**Figure 3:**
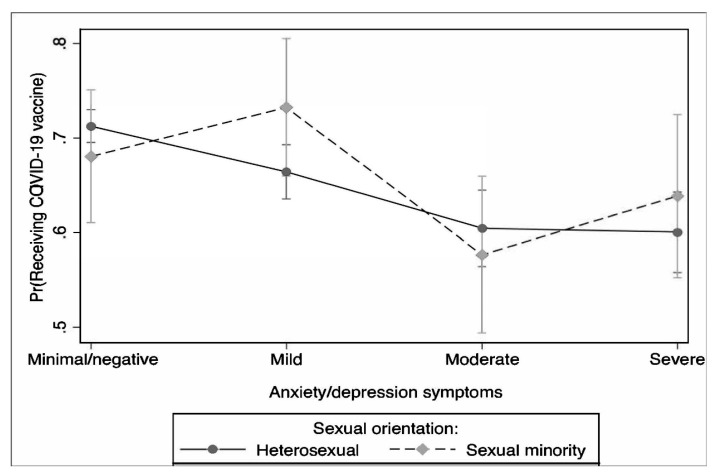
Differences in the perceived likelihood of receiving the COVID-19 vaccine between and within sexual orientation and anxiety/depression symptoms.

Figure 3 shows that the moderation effects of sexual orientation on anxiety/depression symptoms influence the likelihood of receiving the COVID-19 vaccine. Sexual orientation significantly moderated the association between anxiety/depression symptoms and the perceived likelihood of receiving the COVID-19 vaccine (chi2[4] = 47.45, *p* < 0.001). The highest probability of being likely to receive the COVID-19 vaccine was observed among sexual minority individuals who had mild anxiety/depression symptoms.

**Figure 4:**
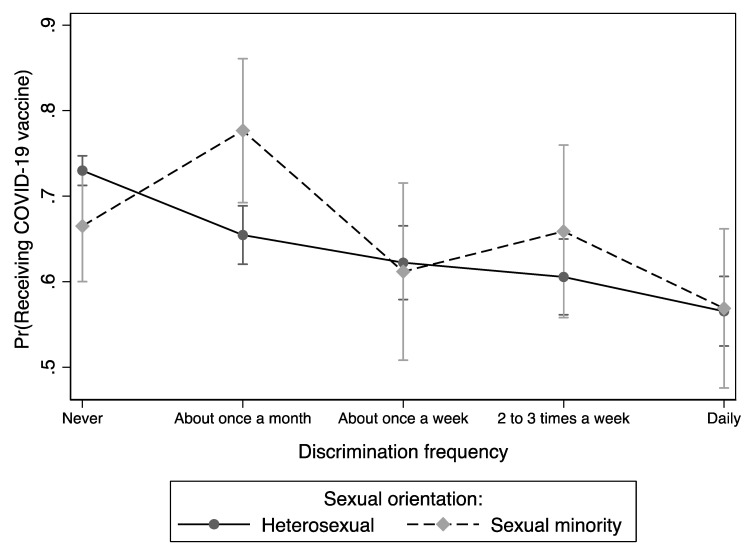
Differences in the perceived likelihood of receiving the COVID-19 vaccine between and within sexual orientation and discrimination frequency during the pandemic.

The moderation effects of sexual orientation on the association between the perceived likelihood of receiving the COVID-19 vaccine and discrimination frequency during the pandemic are presented in Figure 4. Sexual orientation significantly moderated the association between the perceived likelihood of receiving the COVID-19 vaccine and discrimination frequency during the pandemic (chi2[5] = 84.13, *p* < 0.001). Sexual minority individuals who experienced discrimination about once a month during the pandemic had the highest probability of COVID-19 vaccine intention compared to their counterparts.

### 3.2. Multivariate Logistic Regression

Stratified by sexual orientation, the logistic regression analysis results of the associations between the perceived likelihood of receiving the COVID-19 vaccine and sociodemographic characteristics, perceived likelihood of contracting COVID-19, anxiety/depression symptoms, discrimination frequency, and social distancing stress are presented in Table 2.

*Heterosexual participants*: Individuals aged 35–49 (AOR = 1.86, 95% CI = 1.51, 2.30) or 50 or more (AOR = 3.50, 95% CI = 2.41, 5.08) had a higher perceived likelihood of receiving the COVID-19 vaccine compared those aged 18–25 years. The odds were higher for Asians (AOR = 2.21, 95% CI = 1.69, 2.89) and Hispanics/Latinos (AOR = 1.36, 95% CI = 1.13, 1.65) compared to White participants. Lower odds were observed among those with less than High School (AOR = 0.34, 95% CI = 0.25, 0.47), High School diploma or GED (AOR = 0.47, 95% CI = 0.39, 0.57), or some college/vocational or technical school (AOR = 0.59, 95% CI = 0.50, 0.70) compared to those with a college or higher degree; lower odds were observed among those with less than a $25,000 (AOR = 0.57, 95% CI = 0.46, 0.70), $25,000 to <$35,000 (AOR = 0.55, 95% CI = 0.44, 0.69), $35,000 to <$50,000 (AOR = 0.57, 95% CI = 0.46, 0.72), or $50,000 to <$75,000 (AOR = 0.77, 95% CI = 0.62, 0.95) annual household income compared to those with $75,000 or more. Those who had a perceived likelihood of contracting COVID-19 had a higher likelihood of receiving the COVID-19 vaccine (AOR = 1.32, 95% CI = 1.15, 1.53) compared to those who perceived no fear or likelihood of contracting COVID-19. The lower odds were found among individuals who experienced discrimination about once a month (AOR = 0.69, 95% CI = 0.57, 0.84), once a week (AOR = 0.75, 95% CI = 0.60, 0.95), 2 to 3 times a week (AOR = 0.72, 95% CI = 0.57, 0.91), and daily or almost daily (AOR = 0.61, 95% CI = 0.49, 0.76) during the pandemic compared to those who had never experienced discrimination. Compared to those reporting that social distancing was not stressful, those who reported social distancing as stressful had a higher perceived likelihood of receiving the COVID-19 vaccine (AOR = 1.22, 95% CI = 1.06, 1.42). Gender identity and anxiety/depression symptoms were not significantly associated with the perceived likelihood of receiving the COVID-19 vaccine.

*Sexual minority participants*: Compared to individuals aged 18–25 years, those aged 35–49 had a higher perceived likelihood of receiving the COVID-19 vaccine (AOR = 7.00, 95% CI = 2.54, 19.29). Black/African Americans had lower odds (AOR = 0.54, 95% CI = 0.33, 0.91), while Asians had higher odds (AOR = 2.91, 95% CI = 1.17, 7.24) compared to White respondents. Those with less than High School (AOR = 0.33, 95% CI = 0.16, 0.71), High School diploma or GED (AOR = 0.33, 95% CI = 0.18, 0.62), or some college/vocational or technical school (AOR = 0.28, 95% CI = 0.15, 0.52) had a lower perceived likelihood of receiving the COVID-19 vaccine compared to those with a college or higher degree. Individuals who experienced discrimination about once a month during the pandemic had a higher perceived likelihood of receiving the COVID-19 vaccine (AOR = 2.20, 95% CI = 1.15, 4.18) relative to their counterparts who never experienced discrimination. Gender identity, annual household income, perceived likelihood of contracting COVID-19, anxiety/depression symptoms, and social distancing stress did not significantly influence the likelihood of receiving the COVID-19 vaccine.

## 4. Discussion

The present study elucidated interesting trends across sexual orientation for the perceived likelihood of receiving the COVID-19 vaccine. Our multivariate logistic regression analysis yielded similar findings to previous literature as individuals who reported lower income (heterosexual respondents) and education levels (heterosexual and sexual minority respondents) were less likely to report being likely to receive the COVID-19 vaccine [29,30,31,32,33]. This illustrates an important area of concern and intervention for vaccine education and promotion, given individuals of lower socioeconomic status have the highest risk for poor respiratory health [34]. While heterosexual individuals had a higher prevalence of vaccination intention compared to sexual minority participants when aggregated, further analysis of disaggregated sexual orientation showed a higher likelihood of vaccination among gay individuals compared to heterosexual, bisexual, and lesbian participants—with heterosexual participants having a higher prevalence than bisexual and lesbian participants. These findings mirror the variability in vaccination intention across sexual orientations found in previous studies [13,35]. As such, there may be vaccination disparities across sexual orientation groups and, thus, a need to tailor vaccination information, campaigns, and resources to the heterogeneity of sexual minority communities.

Among sexual minority respondents, Black/African American individuals had the lowest likelihood to report vaccination intention, which mirrors prior research [13]. The low likelihood of vaccination among Black/African American individuals may be explained by sexual minority people of color being doubly stigmatized and at risk for discrimination, medical abuse, and harassment due to their sexual orientation and race [36,37,38]. Our finding is particularly concerning given Black sexual minority individuals have also reported a higher prevalence of asthma, diabetes, hypertension, kidney disease, obesity, stroke, and smoking compared to heterosexual persons and other racial/ethnic groups [4]. These comorbidities have also been shown to increase morbidity and mortality among COVID-19-infected patients, especially among Black/African Americans [39,40,41]. Heightened fears of abuse and harassment both inside and outside the clinical setting, coupled with a higher risk of contracting COVID-19, may subsequently negatively impact vaccination coverage. Indeed, Black LGBT individuals have reported lower care access and vaccination coverage compared to other racial/ethnic groups, highlighting an urgent need for more access to healthcare, vaccine education, and vaccines among communities of color [9,13,15,22].

In line with TPB postulations, our moderation analysis showed individuals who reported a higher perceived likelihood of contracting COVID-19 were more likely to express vaccine intent, which is consistent with other studies [20,23,42,43]. Interestingly, we also found sexual minority individuals with mild anxiety/depression symptoms had the highest likelihood of receiving the COVID-19 vaccine, but individuals with moderate and severe anxiety/depression had a lower likelihood of vaccination. Prior research in this area is inconsistent, with some showing individuals with mental health conditions are more likely to be vaccinated [44,45], others showing they are less likely [43,46,47,48], and some showing variability in vaccination by the type and severity of mental health symptoms [44]. We provide evidence of heterogeneity in vaccine intent by sexual orientation and level of anxiety/depression symptoms, illustrating individuals with mild and severe anxiety/depression may be the most at risk for being unvaccinated. Our findings may be a result of the higher severity of anxiety/depression leading to lower self-efficacy [47]. Prior research has also highlighted the higher mental health and economic burden during the pandemic [6,49,50,51,52,53]. Moreover, individuals with mental health conditions, especially sexual minorities, are more likely to lack trust in the medical system, experience stigma, and face socioeconomic and structural barriers to care, meaning lower reported vaccine intent among this population is of urgent concern [47,54,55,56].

Sexual minorities face a multitude of socioeconomic and structural factors that adversely impact their health outcomes, including discrimination and stigma [57,58,59,60]. Indeed, sexual minority persons have reported high levels of discrimination during the pandemic [4,61,62]. In the present study, individuals that reported discrimination once a week or more had the lowest probability of receiving the vaccine for both sexual orientation groups. Avoidance of medical care because of perceived discrimination has been documented among racial/ethnic and sexual minority groups [63,64,65]. Thus, discrimination experiences may also lead to vaccine hesitancy [48], highlighting a need to improve trust and engagement in healthcare settings with populations vulnerable to discrimination. Interestingly, sexual minority individuals who experienced discrimination once a month had the highest perceived probability of receiving the COVID-19 vaccine, indicating a deviation from prior literature [66,67]. Thus, further research is needed to elucidate the mechanism behind this finding.

## 5. Limitations

Even though the data from this cross-sectional survey provided a large sample and valuable information on the likelihood of receiving the COVID-19 vaccine among sexual minorities, it is not representative of the US population. In addition, the cross-sectional nature of the data prevents us from establishing temporal sequence or causality. Also, individuals already vaccinated may have been included in the study due to the fact that the survey was conducted without vaccination status verification. Moreover, the perception or likelihood of receiving the COVID-19 vaccine may not translate into actual vaccination among the survey respondents. The survey was conducted only in English and therefore did not include some populations of the US whose primary language is not English. Furthermore, attitude towards vaccination is very dependent on time. Subsequently, this might have shifted participants’ perception regarding the COVID-19 vaccine, considering the timespan of the data collection (May 2021–January 2022). Lastly, individuals without internet access or a mobile plan might not have been recruited because the survey was conducted online.

## 6. Conclusions

Our findings further underline the disproportionately lower vaccination intention among Black/African American sexual minorities and other vulnerable groups. These findings highlight the need for targeted educational approaches for minority populations on the effectiveness of the vaccine and healthcare providers on the concerns and hesitations of sexual minorities, especially Black/African American sexual minority persons. Additionally, this study further highlights the crucial role of discrimination and anxiety/depression in the high likelihood of receiving the COVID-19 vaccine among both sexual minorities and heterosexual populations. These findings further support the need for the development of creative targeted approaches to understanding and addressing the sources and causes of anxiety/depression and discrimination among these populations to improve vaccination acceptance.

## Figures and Tables

**Table 1:** Descriptive and bivariate analyses of likelihood of receiving COVID-19 vaccine by sociodemographic characteristics, likelihood of contracting COVID-19, anxiety/depression, discrimination, and social distancing stress (N = 5404).

	Heterosexual Participants			Sexual Minority Participants		
	Total n (%)	Likely to Receive COVID-19 Vaccine n (%)	*p*-Value	Total n (%)	Likely to Receive COVID-19 Vaccine n (%)	*p*-Value
**Characteristics**	4797	3241 (67.56)		576	378 (65.62)	
**Age groups**			<0.001			<0.001
18–25	1005 (20.95)	702 (69.85)		90 (15.62)	55 (61.11)	
26–34	666 (13.88)	446 (66.97)		57 (9.90)	41 (71.93)	
35–49	1106 (23.06)	847 (76.58)		73 (12.67)	67 (91.78)	
50 or more	341 (7.11)	299 (87.68)		13 (2.26)	8 (61.54)	
Missing	1679 (35.00)	947 (56.40)		343 (59.55)	207 (60.35)	
**Gender identity**			0.078			0.037
Man	1742 (36.36)	1213 (69.63)		154 (26.74)	114 (74.03)	
Something else/ non-binary/transgender	38 (0.79)	26 (68.42)		78 (13.54)	49 (62.82)	
Woman	3011 (62.85)	2001 (66.46)		344 (59.72)	215 (62.50)	
**Race/ethnicity**			<0.001			<0.001
Another race/ethnicity	178 (3.71)	110 (61.80)		41 (7.12)	22 (53.66)	
Asian	504 (10.51)	413 (81.94)		50 (8.68)	42 (84.00)	
Black/African American	1192 (24.85)	698 (58.56)		143 (24.83)	76 (53.15)	
Hispanic/Latino	848 (17.68)	578 (68.16)		128 (22.22)	89 (69.53)	
White	2075 (43.26)	1442 (69.49)		214 (37.15)	149 (69.63)	
**Level of education completed**			<0.001			<0.001
Less than high school	250 (5.22)	115 (46.00)		65 (11.28)	33 (50.77)	
High school diploma or GED	1074 (22.43)	605 (56.33)		162 (28.12)	92 (56.79)	
Some college/vocational or technical school	1554 (32.46)	1003 (64.54)		177 (30.73)	107 (60.45)	
College or higher degree	1910 (39.89)	1512 (79.16)		172 (29.86)	146 (84.88)	
**Annual household income**			<0.001			<0.001
Less than $25,000	1127 (23.74)	650 (57.68)		171 (29.95)	90 (52.63)	
$25,000 to < $35,000	717 (15.10)	427 (59.55)		100 (17.51)	66 (66.00)	
$35,000 to < $50,000	723 (15.23)	455 (62.93)		98 (17.16)	61 (62.24)	
$50,000 to < $75,000	880 (18.53)	631 (71.70)		106 (18.56)	80 (75.47)	
$75,000 or more	1301 (27.40)	1047 (80.48)		96 (16.81)	76 (79.17)	
**Likelihood of contracting COVID-19**			<0.001			0.021
Not at all likely	1506 (31.45)	946 (62.82)		186 (32.35)	110 (59.14)	
Likely	3283 (68.55)	2290 (69.75)		389 (67.65)	268 (68.89)	
**Anxiety/depression symptoms**			<0.001			0.044
Minimal/negative	2619 (55.42)	1866 (71.25)		169 (29.81)	115 (68.05)	
Mild	1033 (21.86)	686 (66.41)		142 (25.04)	104 (73.24)	
Moderate	566 (11.98)	342 (60.42)		137 (24.16)	79 (57.66)	
Severe	508 (10.75)	305 (60.04)		119 (20.99)	76 (63.87)	
**Discrimination frequency**			<0.001			0.032
Never	2521 (52.59)	1840 (72.99)		203 (35.24)	135 (66.50)	
About once a month	744 (15.52)	487 (65.46)		94 (16.32)	73 (77.66)	
About once a week	487 (10.16)	303 (62.22)		85 (14.76)	52 (61.18)	
2 to 3 times a week	469 (9.78)	284 (60.55)		85 (14.76)	56 (65.88)	
Daily or almost daily	573 (11.95)	324 (56.54)		109 (18.92)	62 (56.88)	
**Social distancing stress**			0.58			0.79
Not at all stressful	1525 (31.84)	1002 (65.70)		149 (25.87)	89 (59.73)	
Stressful	3265 (68.16)	2235 (68.45)		427 (74.13)	289 (67.68)	

Note: Differences in total numbers in categories may be due to missing data.

**Table 2:** Multivariable logistic regression analysis of the associations between the likelihood of receiving a COVID-19 vaccine and sociodemographic characteristics, the fear or likelihood of contracting COVID-19, anxiety/depression, discrimination, and social distancing stress across sexual identity populations.

	Heterosexual Participants		Sexual Minority Participants	
	AOR	95% CI	AOR	95% CI
**Age groups**				
18–25	Ref	-	Ref	-
26–34	1.08	(0.86, 1.36)	1.58	(0.70, 3.53)
35–49	1.86 ***	(1.51, 2.30)	7.00 ***	(2.54, 19.29)
50 or more	3.50 ***	(2.41, 5.08)	0.77	(0.19, 3.17)
Missing	0.74 **	(0.62, 0.89)	0.97	(0.55, 1.71)
**Gender identity**				
Man	1.13	(0.98, 1.30)	1.15	(0.68, 1.95)
Something else/non-binary/transgender	1.49	(0.69, 3.22)	1.18	(0.65, 2.15)
Women	Ref	-	Ref	-
**Race/ethnicity**				
Asian	2.21 ***	(1.69, 2.89)	2.91 *	(1.17, 7.24)
Black/African American	0.89	(0.76, 1.06)	0.54 *	(0.33, 0.91)
Hispanic/Latino	1.36 **	(1.13, 1.65)	1.19	(0.69, 2.05)
White	Ref	-	Ref	-
**Level of education completed**				
Less than high school	0.34 ***	(0.25, 0.47)	0.33 **	(0.16, 0.71)
High school diploma or GED	0.47 ***	(0.39, 0.57)	0.33 ***	(0.18, 0.62)
Some college/vocational or technical school	0.59 ***	(0.50, 0.70)	0.28 ***	(0.15, 0.52)
College or higher degree	Ref	-	Ref	-
**Annual household income**				
Less than $25,000	0.57 ***	(0.46, 0.70)	0.51	(0.26, 1.02)
$25,000 to < $35,000	0.55 ***	(0.44, 0.69)	0.99	(0.47, 2.08)
$35,000 to < $50,000	0.57 ***	(0.46, 0.72)	0.67	(0.32, 1.40)
$50,000 to < $75,000	0.77 *	(0.62, 0.95)	1.33	(0.62, 2.84)
$75,000 or more	Ref	-	Ref	-
**Likelihood of contracting COVID-19**				
Not at all likely	Ref	-	Ref	-
Likely	1.32 ***	(1.15, 1.53)	1.22	(0.80, 1.87)
**Anxiety/depression symptoms**				
Minimal/negative	Ref	-	Ref	-
Mild	1.12	(0.94, 1.34)	1.76	(0.96, 3.22)
Moderate	1.03	(0.83, 1.29)	0.84	(0.47, 1.52)
Severe	1.04	(0.83, 1.31)	1.08	(0.58, 2.01)
**Discrimination frequency**				
Never	Ref	-	Ref	-
About once a month	0.69 ***	(0.57, 0.84)	2.20 *	(1.15, 4.18)
About once a week	0.75 *	(0.60, 0.95)	0.77	(0.41, 1.44)
2 to 3 times a week	0.72 **	(0.57, 0.91)	1.34	(0.70, 2.59)
Daily or almost daily	0.61 ***	(0.49, 0.76)	0.93	(0.51, 1.68)
**Social distancing stress**				
Not at all stressful	Ref	-	Ref	-
Stressful	1.22 **	(1.06, 1.42)	1.11	(0.69, 1.79)

AOR = Adjusted odds ratio. 95% CI = 95% confidence interval. Ref = Reference group. * *p* < 0.05, ** *p* < 0.01, *** *p* < 0.001.

## Data Availability

Data is available upon request.

## References

[1] Webb Hooper M., Nápoles A.M., Pérez-Stable E.J. (2020). COVID-19 and Racial/Ethnic Disparities. JAMA.

[2] Lopez L., Hart L.H., Katz M.H. (2021). Racial and Ethnic Health Disparities Related to COVID-19. JAMA.

[3] Mackey K., Ayers C.K., Kondo K.K., Saha S., Advani S.M., Young S., Spencer H., Rusek M., Anderson J., Veazie S. (2020). Racial and Ethnic Disparities in COVID-19–Related Infections, Hospitalizations, and Deaths. Ann. Intern. Med..

[4] Heslin K.C., Hall J.E. (2021). Sexual Orientation Disparities in Risk Factors for Adverse COVID-19-Related Outcomes, by Race/Ethnicity–Behavioral Risk Factor Surveillance System, United States, 2017–2019. MMWR Morb. Mortal Wkly. Rep..

[5] Bragazzi N.L. (2021). The COVID-19 Pandemic Seen from a Syndemic Perspective: The LGBTQIA2SP+ Community. Infect. Dis. Rep..

[6] Dawson L., Kirzinger A., Kates J. (2021). The Impact of the COVID-19 Pandemic on LGBT People.

[7] Andraska E.A., Alabi O., Dorsey C., Erben Y., Velazquez G., Franco-Mesa C., Sachdev U. (2021). Health care disparities during the COVID-19 pandemic. Semin. Vasc. Surg..

[8] Fish J.N., Salerno J., Williams N.D., Rinderknecht R.G., Drotning K.J., Sayer L., Doan L. (2021). Sexual Minority Disparities in Health and Well-Being as a Consequence of the COVID-19 Pandemic Differ by Sexual Identity. LGBT Health.

[9] Phillips Ii G., Xu J., Ruprecht M.M., Costa D., Felt D., Wang X., Glenn E.E., Beach L.B. (2021). Associations with COVID-19 Symptoms, Prevention Interest, and Testing Among Sexual and Gender Minority Adults in a Diverse National Sample. LGBT Health.

[10] Hughes L., Shireman T.I., Hughto J. (2021). Privately Insured Transgender People Are At Elevated Risk For Chronic Conditions Compared With Cisgender Counterparts. Health Aff..

[11] Rich A.J., Scheim A.I., Koehoorn M., Poteat T. (2020). Non-HIV chronic disease burden among transgender populations globally: A systematic review and narrative synthesis. Prev. Med. Rep..

[12] Nowaskie D.Z., Roesler A.C. (2022). The impact of COVID-19 on the LGBTQ+ community: Comparisons between cisgender, heterosexual people, cisgender sexual minority people, and gender minority people. Psychiatry Res..

[13] McNaghten A.D., Brewer N.T., Hung M.C., Lu P.J., Daskalakis D., Abad N., Kriss J., Black C., Wilhelm E., Lee J.T. (2022). COVID-19 Vaccination Coverage and Vaccine Confidence by Sexual Orientation and Gender Identity *–* United States, 29 August–30 October 2021. MMWR Morb. Mortal Wkly. Rep..

[14] Baćak V., Thurman K., Eyer K., Qureshi R., Bird J.D.P., Rivera L.M., Kim S.A. (2018). Incarceration as a Health Determinant for Sexual Orientation and Gender Minority Persons. Am. J. Public Health.

[15] Ruprecht M.M., Wang X., Johnson A.K., Xu J., Felt D., Ihenacho S., Stonehouse P., Curry C.W., DeBroux C., Costa D. (2021). Evidence of Social and Structural COVID-19 Disparities by Sexual Orientation, Gender Identity, and Race/Ethnicity in an Urban Environment. J. Urban Health.

[16] Macapagal K., Bhatia R., Greene G.J. (2016). Differences in Healthcare Access, Use, and Experiences Within a Community Sample of Racially Diverse Lesbian, Gay, Bisexual, Transgender, and Questioning Emerging Adults. LGBT Health.

[17] Ormiston C.K., Williams F. (2022). LGBTQ youth mental health during COVID-19: Unmet needs in public health and policy. Lancet.

[18] Sell R.L., Krims E.I. (2021). Structural Transphobia, Homophobia, and Biphobia in Public Health Practice: The Example of COVID-19 Surveillance. Am. J. Public Health.

[19] Quinn K.G., Walsh J.L., John S.A., Nyitray A.G. (2021). “I Feel Almost as Though I’ve Lived This Before”: Insights from Sexual and Gender Minority Men on Coping with COVID-19. AIDS Behav..

[20] Azucar D., Slay L., Valerio D.G., Kipke M.D. (2022). Barriers to COVID-19 Vaccine Uptake in the LGBTQIA Community. Am. J. Public Health.

[21] Teixeira da Silva D., Biello K., Lin W.Y., Valente P.K., Mayer K.H., Hightow-Weidman L., Bauermeister J.A. (2021). COVID-19 Vaccine Acceptance among an Online Sample of Sexual and Gender Minority Men and Transgender Women. Vaccines.

[22] Yasmin F., Najeeb H., Moeed A., Naeem U., Asghar M.S., Chughtai N.U., Yousaf Z., Seboka B.T., Ullah I., Lin C.Y. (2021). COVID-19 Vaccine Hesitancy in the United States: A Systematic Review. Front. Public Health.

[23] Limbu Y.B., Gautam R.K., Zhou W. (2022). Predicting Vaccination Intention against COVID-19 Using Theory of Planned Behavior: A Systematic Review and Meta-Analysis. Vaccines.

[24] The Trevor Project The Spectrum. https://www.thetrevorproject.org/wp-content/uploads/2017/09/Spectrum-B.pdf.

[25] Kroenke K., Spitzer R.L., Williams J.B.W., Löwe B. (2009). An Ultra-Brief Screening Scale for Anxiety and Depression: The PHQ–4. Psychosomatics.

[26] Löwe B., Wahl I., Rose M., Spitzer C., Glaesmer H., Wingenfeld K., Schneider A., Brähler E. (2010). A 4-item measure of depression and anxiety: Validation and standardization of the Patient Health Questionnaire-4 (PHQ-4) in the general population. J. Affect. Disord..

[27] Bayman E.O., Dexter F. (2021). Multicollinearity in Logistic Regression Models. Anesth. Analg..

[28] Stata Technical Support (2019). Stata Statistical Software.

[29] Ta Park V.M., Dougan M., Meyer O.L., Nam B., Tzuang M., Park L.G., Vuong Q., Tsoh J.Y. (2021). Vaccine willingness: Findings from the COVID-19 effects on the mental and physical health of Asian Americans & Pacific Islanders survey study (COMPASS). Prev. Med. Rep..

[30] Khubchandani J., Sharma S., Price J.H., Wiblishauser M.J., Sharma M., Webb F.J. (2021). COVID-19 Vaccination Hesitancy in the United States: A Rapid National Assessment. J. Community Health.

[31] Latkin C., Dayton L., Miller J., Yi G., Balaban A., Boodram B., Uzzi M., Falade-Nwulia O. (2022). A longitudinal study of vaccine hesitancy attitudes and social influence as predictors of COVID-19 vaccine uptake in the US. Hum. Vaccines Immunother..

[32] Samoa R.A., Ðoàn L.N., Saw A., Aitaoto N., Takeuchi D. (2022). Socioeconomic Inequities in Vaccine Hesitancy Among Native Hawaiians and Pacific Islanders. Health Equity.

[33] Gutierrez S., Logan R., Marshall C., Kerns J., Diamond-Smith N. (2022). Predictors of COVID-19 Vaccination Likelihood Among Reproductive-Aged Women in the United States. Public Health Rep..

[34] Gaffney A.W., Himmelstein D.U., Christiani D.C., Woolhandler S. (2021). Socioeconomic Inequality in Respiratory Health in the US From 1959 to 2018. JAMA Intern. Med..

[35] Low A., Wright C., Platt J., Chang C., Mantell J.E., Romero E., Hoos D., Mannheimer S., Greenleaf A., Castor D. (2022). COVID-19 Vaccine Uptake and Factors Associated With Being Unvaccinated Among Lesbian, Gay, Bisexual, Transgender, Queer, and Other Sexual Identities (LGBTQ+) New Yorkers. Open Forum Infect. Dis..

[36] Brito N.H., Werchan D., Brandes-Aitken A., Yoshikawa H., Greaves A., Zhang M. (2022). Paid maternal leave is associated with infant brain function at 3 months of age. Child Dev..

[37] Cahill S., Grasso C., Keuroghlian A., Sciortino C., Mayer K. (2020). Sexual and Gender Minority Health in the COVID-19 Pandemic: Why Data Collection and Combatting Discrimination Matter Now More Than Ever. Am. J. Public Health.

[38] Swann G., Stephens J., Newcomb M.E., Whitton S.W. (2020). Effects of sexual/gender minority- and race-based enacted stigma on mental health and substance use in female assigned at birth sexual minority youth. Cult. Divers. Ethn. Minor. Psychol..

[39] Kompaniyets L., Pennington A.F., Goodman A.B., Rosenblum H.G., Belay B., Ko J.Y., Chevinsky J.R., Schieber L.Z., Summers A.D., Lavery A.M. (2021). Underlying Medical Conditions and Severe Illness Among 540,667 Adults Hospitalized With COVID-19, March 2020–March 2021. Prev. Chronic Dis..

[40] Li X., Zhong X., Wang Y., Zeng X., Luo T., Liu Q. (2021). Clinical determinants of the severity of COVID-19: A systematic review and meta-analysis. PLoS ONE.

[41] Nguyen H., Medina A., Golovko G., Evangelista L. (2022). Racial and Ethnic Differences in Fatality Risk From COVID-19. SAGE Open Nurs..

[42] Weinstein E.R., Balise R., Metheny N., Jose Baeza Robba M., Mayo D., Michel C., Chan B., Safren S.A., Harkness A. (2022). Factors associated with latino sexual minority men’s likelihood and motivation for obtaining a COVID-19 vaccine: A mixed-methods study. J. Behav. Med..

[43] Sekizawa Y., Hashimoto S., Denda K., Ochi S., So M. (2022). Association between COVID-19 vaccine hesitancy and generalized trust, depression, generalized anxiety, and fear of COVID-19. BMC Public Health.

[44] Hassan L., Sawyer C., Peek N., Lovell K., Carvalho A.F., Solmi M., Tilston G., Sperrin M., Firth J. (2022). COVID-19 vaccination uptake in people with severe mental illness: A UK-based cohort study. World Psychiatry.

[45] Bendau A., Plag J., Petzold M.B., Ströhle A. (2021). COVID-19 vaccine hesitancy and related fears and anxiety. Int. Immunopharmacol..

[46] Nguyen K.H., Chen S., Morris K., Chui K., Allen J.D. (2022). Mental health symptoms and association with COVID-19 vaccination receipt and intention to vaccinate among adults, United States. Prev. Med..

[47] Eyllon M., Dang A.P., Barnes J.B., Buresh J., Peloquin G.D., Hogan A.C., Shimotsu S.T., Sama S.R., Nordberg S.S. (2022). Associations between psychiatric morbidity and COVID-19 vaccine hesitancy: An analysis of electronic health records and patient survey. Psychiatry Res..

[48] Adzrago D., Sulley S., Ormiston C.K., Mamudu L., Williams F. (2022). Differences in the Perceived Likelihood of Receiving COVID-19 Vaccine. Int. J. Environ. Res. Public Health.

[49] Wojcik H., Breslow A.S., Fisher M.R., Rodgers C.R.R., Kubiszewski P., Gabbay V. (2022). Mental Health Disparities Among Sexual and Gender Minority Frontline Health Care Workers During the Height of the COVID-19 Pandemic. LGBT Health.

[50] Waters A.R., Bybee S., Warner E.L., Kaddas H.K., Kent E.E., Kirchhoff A.C. (2022). Financial Burden and Mental Health Among LGBTQIA+ Adolescent and Young Adult Cancer Survivors During the COVID-19 Pandemic. Front. Oncol..

[51] MacCarthy S., Izenberg M., Barreras J.L., Brooks R.A., Gonzalez A., Linnemayr S. (2020). Rapid mixed-methods assessment of COVID-19 impact on Latinx sexual minority men and Latinx transgender women. PLoS ONE.

[52] Racine N., McArthur B.A., Cooke J.E., Eirich R., Zhu J., Madigan S. (2021). Global Prevalence of Depressive and Anxiety Symptoms in Children and Adolescents During COVID-19: A Meta-analysis. JAMA Pediatr..

[53] Ettman C.K., Abdalla S.M., Cohen G.H., Sampson L., Vivier P.M., Galea S. (2020). Prevalence of Depression Symptoms in US Adults Before and During the COVID-19 Pandemic. JAMA Netw. Open.

[54] Roulston C., McKetta S., Price M., Fox K.R., Schleider J.L. (2022). Structural Correlates of Mental Health Support Access among Sexual Minority Youth of Color during COVID-19. J. Clin. Child Adolesc. Psychol..

[55] Whaibeh E., Mahmoud H., Vogt E.L. (2020). Reducing the Treatment Gap for LGBT Mental Health Needs: The Potential of Telepsychiatry. J. Behav. Health Serv. Res..

[56] Williams N.D., Fish J.N. (2020). The availability of LGBT-specific mental health and substance abuse treatment in the United States. Health Serv. Res..

[57] Mattocks K.M., Sullivan J.C., Bertrand C., Kinney R.L., Sherman M.D., Gustason C. (2015). Perceived Stigma, Discrimination, and Disclosure of Sexual Orientation Among a Sample of Lesbian Veterans Receiving Care in the Department of Veterans Affairs. LGBT Health.

[58] Jackson C.L., Agénor M., Johnson D.A., Austin S.B., Kawachi I. (2016). Sexual orientation identity disparities in health behaviors, outcomes, and services use among men and women in the United States: A cross-sectional study. BMC Public Health.

[59] Institute of Medicine (U.S.) (2011). Committee on Lesbian Gay Bisexual and Transgender Health Issues and Research Gaps and Opportunities. The Health of Lesbian, Gay, Bisexual, and Transgender People: Building a Foundation for Better Understanding.

[60] Patterson C., Sepuúlveda M.n.-J., White J., National Academies of Sciences Engineering and Medicine (U.S.) (2020). Committee on Understanding the Well-Being of Sexual and Gender Diverse Populations; National Academies of Sciences Engineering and Medicine (U.S.); Committee on Population. Understanding the Well-Being of LGBTQI+ Populations. Consens. Study Rep. Natl. Acad. Sci. Eng. Med..

[61] Kneale D., Bécares L. (2021). Discrimination as a predictor of poor mental health among LGBTQ+ people during the COVID-19 pandemic: Cross-sectional analysis of the online Queerantine study. BMJ Open.

[62] Apodaca C., Casanova-Perez R., Bascom E., Mohanraj D., Lane C., Vidyarthi D., Beneteau E., Sabin J., Pratt W., Weibel N. (2022). Maybe they had a bad day: How LGBTQ and BIPOC patients react to bias in healthcare and struggle to speak out. J. Am. Med. Inform. Assoc..

[63] Holder-Dixon A.R., Adams O.R., Cobb T.L., Goldberg A.J., Fikslin R.A., Reinka M.A., Gesselman A.N., Price D.M. (2022). Medical avoidance among marginalized groups: The impact of the COVID-19 pandemic. J. Behav Med..

[64] D’Anna L.H., Hansen M., Mull B., Canjura C., Lee E., Sumstine S. (2018). Social Discrimination and Health Care: A Multidimensional Framework of Experiences among a Low-Income Multiethnic Sample. Soc. Work Public Health.

[65] Mensinger J.L., Tylka T.L., Calamari M.E. (2018). Mechanisms underlying weight status and healthcare avoidance in women: A study of weight stigma, body-related shame and guilt, and healthcare stress. Body Image.

[66] Savoia E., Piltch-Loeb R., Goldberg B., Miller-Idriss C., Hughes B., Montrond A., Kayyem J., Testa M.A. (2021). Predictors of COVID-19 Vaccine Hesitancy: Socio-Demographics, Co-Morbidity, and Past Experience of Racial Discrimination. Vaccines.

[67] Garg I., Hanif H., Javed N., Abbas R., Mirza S., Javaid M.A., Pal S., Shekhar R., Sheikh A.B. (2021). COVID-19 Vaccine Hesitancy in the LGBTQ+ Population: A Systematic Review. Infect. Dis. Rep..

